# Frequency and characteristics of pulmonary nodules in children at computed tomography

**DOI:** 10.1007/s00247-017-3946-2

**Published:** 2017-09-04

**Authors:** Atia Samim, Annemieke S. Littooij, Marry M. van den Heuvel-Eibrink, Frank J. Wessels, Rutger A. J. Nievelstein, Pim A. de Jong

**Affiliations:** 1 Department of Radiology, University Medical Centre Utrecht/Wilhelmina Children’s Hospital, HP E01.132, Heidelberglaan 100, 3584 CX Utrecht, The Netherlands; 2 Department of Pediatric Oncology, Princess Máxima Centre for Pediatric Oncology, Lundlaan 6, 3584 EA Utrecht, The Netherlands

**Keywords:** Child, Detection rate, Lungs, Multidetector computed tomography, Observer variability, Pulmonary nodule

## Abstract

**Background:**

Normative data on pulmonary nodules in children without malignancy are limited. Knowledge of the frequency and characteristics of pulmonary nodules in healthy children can influence care decisions in children with malignant disease.

**Objective:**

To provide normative data concerning the frequency and characteristics of pulmonary nodules on computed tomography (CT) in young children.

**Materials and methods:**

All children ages 1 year–12 years who underwent chest CT after high-energy trauma were retrospectively investigated. Exclusion criteria were a history of malignancy, thick image slices, motion artefacts and extensive post-traumatic pulmonary changes. Two radiologists were asked to independently identify all nodules and to characterize each nodule with respect to location, size, perifissural location and calcification. Discrepancies were adjudicated by a third reader, who set the reference standard in this study. Interobserver agreement in detection and characterization was assessed using the kappa coefficient (κ).

**Results:**

Identified were 120 patients, of whom 72 (75% male; median age: 8.0 years [interquartile range: 4–11]) were included. A total of 59 pulmonary nodules were present in 27 patients (38%; 95% confidence interval: 26–49%; range: 1–5 nodules per patient, with a mean diameter of 3.2 mm [standard deviation: 0.9 mm]). For nodule detection, the per-patient interobserver agreement was substantial (*κ*=0.78) and per-lobe agreement was moderate (*κ*=0.40). For characterization, there was fair to substantial agreement (*κ*=0.36–0.74).

**Conclusion:**

Small pulmonary nodules on chest CT are a common finding in otherwise healthy children, but detection and characterization have only moderate interobserver agreement.

## Introduction

A pulmonary nodule in a child with known malignancy can be an important sign of metastatic stage. However, the prevalence and characteristics of nodules in children without malignancy are unclear. Knowledge of these normal values can influence future care decisions in children presenting with a malignancy.

In children, tumours that most frequently disseminate to the lungs are Wilms tumour (nephroblastoma), osteosarcoma and Ewing sarcoma [1, 2]. According to the latest International Society of Paediatric Oncology (SIOP) protocol for renal tumours, a computed tomography (CT) scan of the chest is now mandatory to assess lung metastases [3]. While current CT imaging is highly sensitive in detecting small pulmonary nodules, specificity for metastatic disease is low [4]. In fact, 32% to 65% of the metastasis-suspect nodules on CT in children with various malignancies were proven to be benign at biopsy [5–8]. To prevent unnecessary upstaging due to false-positive diagnosis, correct recognition of benign nodules is important. Despite various attempts in previous literature, it remains difficult to identify CT features that can reliably predict the nature of pulmonary nodules in children with malignancy [5–9]. From studies in adults, some clear clues of benignity have been proposed: Calcified nodules in non-bone-forming tumours would be consistent with granulomas and triangular perifissural nodules with benign intrapulmonary lymph nodes [4]. With regard to the SIOP protocol, international consensus has been reached that cases with nodules ≥3 mm depleted of clear benign characteristics are regarded as malignant.

However, in healthy children, the occurrence of pulmonary nodules without the characteristics associated with benignity is unknown. If nodules ≥3 mm are prevalent in healthy children, there would be a considerable possibility of upstaging in children presenting with Wilms tumour. Furthermore, whether perifissural nodules occur in children has yet to be determined. Another important issue is the inevitable interobserver variability in detecting, measuring and interpreting small pulmonary nodules on CT [10]. Given poor patient compliance, high respiratory rates and limited temporal resolution at chest CT, it is questionable how capable we are at detecting such small lesions.

Only limited literature is available on the prevalence of pulmonary nodules in healthy children. The prevalence of such nodules remains uncertain, as previous studies in children up to the age of 18 years reveal a wide variance, i.e. 33% and 75% [11, 12]. In view of the new SIOP protocol, the prevalence in the younger age groups is particularly relevant, as nearly all cases of Wilms tumour present before the age of 15 years (median: 3.5 years) [13].

By providing the a priori probability of identifying a lung nodule in a child without malignant disease, we aim to improve the estimation of risk of pulmonary metastatic disease. The purpose of this study is to determine the frequency and characteristics of pulmonary nodules and to evaluate interobserver agreement for detecting and characterizing pulmonary nodules in otherwise healthy young children who received a CT scan following trauma.

## Materials and methods

Institutional Review Board (IRB 15–445) approval was obtained. For this retrospective study, formal consent was not required. All procedures performed were in accordance with the ethical standards of the institutional and/or national research committee and with the 1964 Helsinki declaration and its later amendments or comparable ethical standards.

We examined a cohort of consecutive trauma patients as a representation of the healthy paediatric population. All potentially eligible patients from the age of 1 year to 12 years who underwent chest CT as part of their trauma evaluation in a single centre between March 2007 and July 2015 were retrospectively identified. Exclusion criteria were a history of malignancy, scans with ≥1.25-mm slice thickness, diagnostic relevant artefacts and extensive post-traumatic pulmonary changes. Tuberculosis and histoplasmosis are not endemic in our region.

CT examinations were obtained with 16-, 64- and 128-row multidetector CT scanners (Mx8000 IDT 16, Brilliance 16P with 0.75 mm collimation; Brilliance 64, Brilliance iCT with 0.625 mm collimation; Philips Medical Systems, Cleveland, OH, USA). All patients received 2 ml/kg contrast medium according to our trauma protocol. Exposure settings (range: 35–190 mAs and 80–120 kVp) were adjusted to patient size. Thin slice images (1.0-mm maximum thickness at 1.0-mm maximum intervals) were reconstructed, stored in a 512×512 data matrix and displayed with lung parenchyma setting (window level: −500, window width: 1,500).

Two radiologists independently reviewed images of the chest CT of all patients, whilst being aware the study concerned trauma patients. Reader 1 (P.A.d.J.) has >10 years of experience in paediatric and thoracic imaging and reader 2 (F.J.W.) has 5 years of experience in general radiology. Axial thin slice images were available for review, as well as maximum intensity projection (MIP) in all planes to increase sensitivity. The readers recorded the presence of each nodule together with its lobar location (right upper lobe, right middle lobe, right lower lobe, left upper lobe [lingula included] and left lower lobe) and characteristics (nodule size, perifissural [yes/no] and calcification [yes/no]). Nodule size was measured along the long axis diameter at the axial thin slices without the use of MIP. Bone window was used to assess calcification. In case of discrepancy between the two readers, a 3rd reader with >10 years of experience as a paediatric radiologist adjudicated by accepting or rejecting the discrepancies in potential nodules or characteristics and provided a reference standard for our study.

**Fig. 1:**
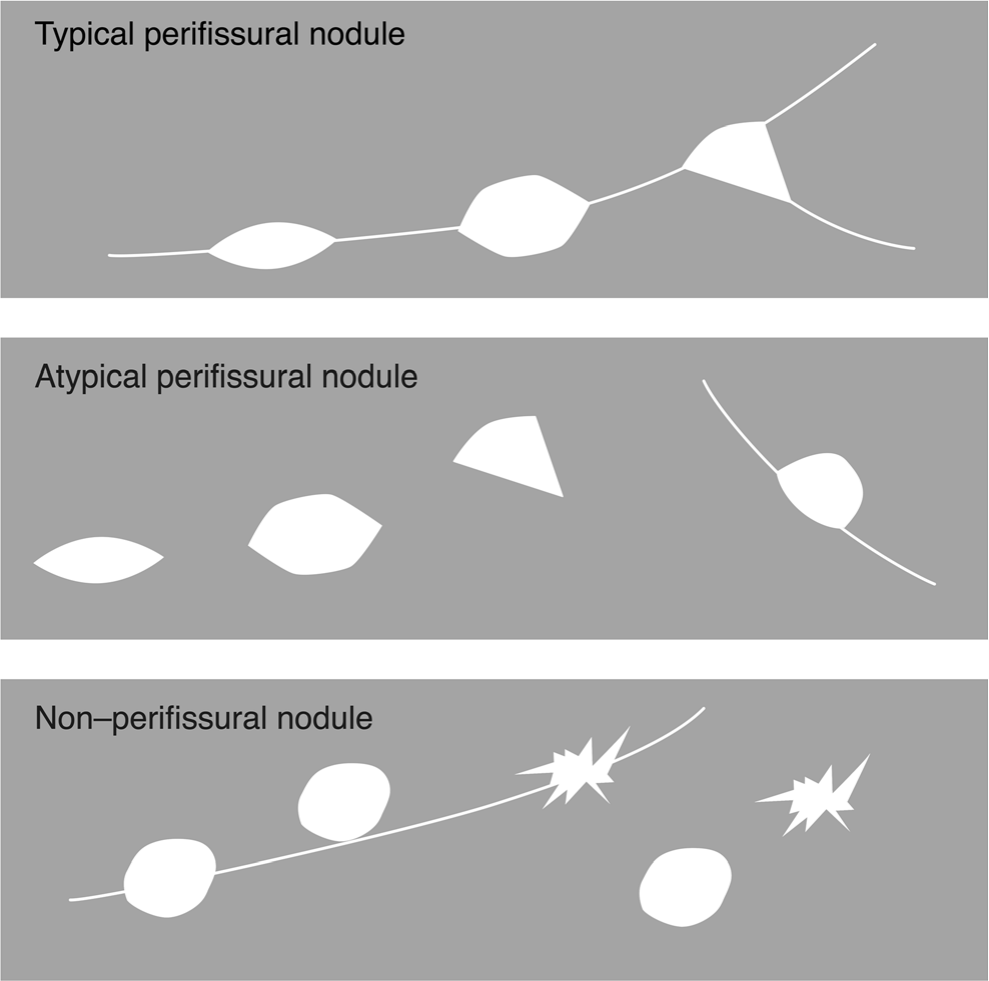
The role of fissure attachment and shape in the classification of perifissural nodules. Reprinted with permission: de Hoop B, den Ginneken B, Gietema H et al., Radiology (2012), © Radiological Society of North America. Fissure-attached nodules with a lentiform, oval or triangular shape are shown in the top pane. Nodules with a lentiform, oval or triangular shape, but not attached to a (visible) fissure *or* fissure attached, convex on one side and rounded on the other are shown in the middle pane. Fissure-(un)attached nodules with a spherical or irregular shape that did not appear to be influenced by the fissure are shown in the bottom pane

Pulmonary nodules were defined as more or less round well-circumscribed opacities in the lung measuring up to 3 cm [4]. De Hoop et al. [14] proposed a classification for perifissural nodules, including the characteristic morphological features of intrapulmonary lymph nodes (Fig. 1). Typically, perifissural nodules appear attached to fissures, have a homogenous solid appearance and a lentiform, oval or triangular shape with smooth margins. However, atypical perifissural nodules also occur; therefore, we chose to dichotomize between perifissural yes (typical and atypical perifissural) and no (non–perifissural nodules).

Numerical variables were summarized, using either mean with standard deviation (SD) or median with interquartile range (IQR), depending on the distribution. All 95% confidence intervals (CI) were calculated using binomial distribution. The interobserver agreement of the detection of pulmonary nodules was assessed in patient-based and lobe-based analyses, which meant that a patient or lobe was marked as positive if the observer found at least one nodule in the patient or lobe. Agreement for characterization was calculated for each individual nodule. Agreement was calculated with the percent agreement and unweighted Cohen’s kappa (κ) statistic, with κ<0 classified as poor, 0.00–0.20 as slight, 0.21–0.40 as fair, 0.41–0.60 as moderate, 0.61–0.80 as substantial and 0.81–1.00 as almost perfect agreement [15]. Nodule frequency is the percentage of patients with at least one nodule among the study sample. We performed logistic regression with nodule presence as the outcome and age as the determinant. Due to the relatively long sampling period, we used logistic regression to explore the effect of the year of the scan on the presence of nodules and linear regression for number of nodules detected and size of the smallest nodule detected. The nodules were put into size categories of smaller nodules (<3 mm) and larger nodules (≥3 mm), based on SIOP’s threshold size for pulmonary metastasis. Multiple nodules were defined as ≥4 nodules per patient. All statistical analyses were performed using SPPS version 21 (SPSS Inc., Chicago, IL, USA). Statistical significance level was set at *P*<0.05.

## Results

### Patients

**Fig. 2:**
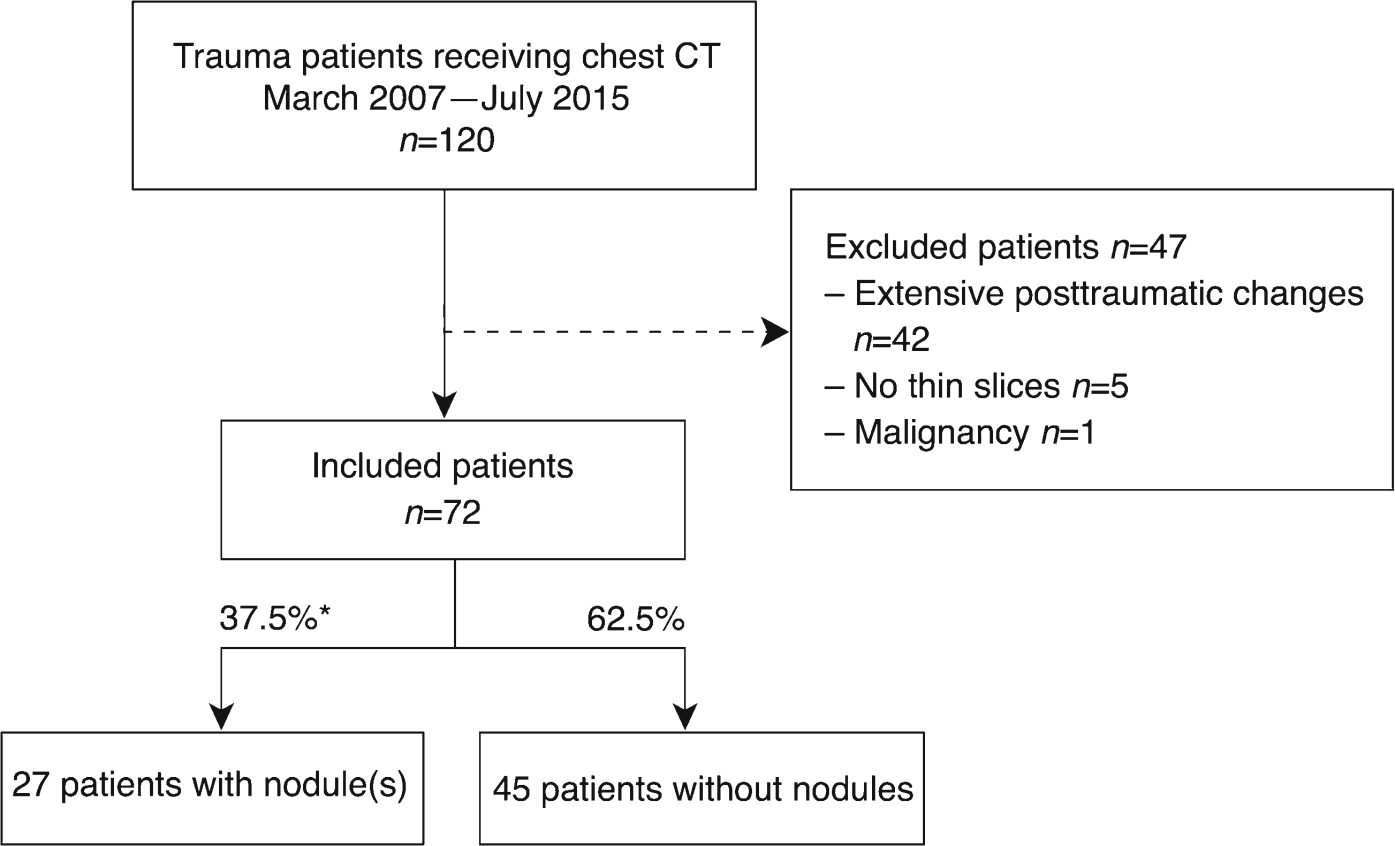
Flow diagram of patient selection. * 95% confidence interval 26–49%, stratification of included patients into subgroups with or without nodules according to the consensus reading

We identified 120 children who underwent chest CT as part of their trauma evaluation. Inclusion and exclusion criteria are illustrated in the flow diagram in Fig. 2. The most frequent reason for exclusion was extensive post-traumatic pleuropulmonary changes (*n=*42), such as aspiration, pneumothorax, pulmonary haemorrhage, contusion and laceration. In five patients, no thin slices were available and one patient was excluded due to a history of lymphoma. This resulted in a study sample of 72 patients (54 males) with a median age of 8.0 (IQR: 4–11) years and CT scans with a median slice thickness of 0.9 mm (range: 0.8–1.0 mm) at 0.5– to 0.8–mm intervals.

### Interobserver agreement

Reader 1 and reader 2 detected 43 and 44 potential nodules, respectively. However, only 22 potential nodules were detected by both readers and the remaining 43 potential nodules were detected by only one reader. Regarding nodule detection, interobserver agreement on a per-patient-basis was substantial (*κ*=0.78), (Table 1). In contrast, agreement on a per-lobe basis was moderate (*κ*=0.40). Regarding characterization, agreement was substantial (*κ*=0.74) for size category, fair (*κ*=0.36) for perifissural location and moderate (*κ*=0.46) for calcification. The consensus reader rejected 6 of the 43 discrepancies in potential nodules, which resulted in a total of 59 nodules that were considered valid. In 18 children with one or more nodules ≥3–5 mm, the nodule was detected by one reader but missed by the other. In two children with one or more nodules ≥5 mm, the nodule was detected by one reader and missed by the other.

**Table 1:** Interobserver agreement using percent agreement and Cohen’s kappa (κ) statistics (95% confidence intervals [CI])

	Reader 1 vs. reader 2		Reader 1 vs. consensus reader		Reader 2 vs. consensus reader	
	% agreement	κ (CI)	% agreement	κ (CI)	% agreement	κ (CI)
Nodule detection						
Patient-based	90% (65/72)^a^	0.78 (0.77–0.80)	94% (68/72)	0.88 (0.86–0.89)	96% (69/72)	0.91 (0.90–0.92)
Lobe-based	89% (319/360)^b^	0.40 (0.25–0.55)	99% (357/360)	0.82 (0.73–0.91)	99% (356/360)	0.75 (0.64–0.86)
Characteristics						
Size category	91% (20/22)	0.74 (0.67–0.82)	93% (38/41)	0.76 (0.71–0.80)	100% (40/40)	1.00 (1.00–1.00)
Perifissural location	68% (15/22)	0.36 (0.29–0.44)	95% (39/41)	0.90 (0.88–0.92)	88% (35/40)	0.70 (0.66–0.74)
Calcification	91% (20/22)	0.46 (0.33–0.60)	100% (41/41)	1.00 (1.00–1.00)	95% (38/40)	0.48 (0.38–0.58)

^a^In 21 patients, both observers detected a nodule, in 44 patients both observers did not find any nodule and in 7 patients one of the observers detected a nodule

^b^In 17 lobes, both observers detected a nodule; in 302 lobes, neither detected a nodule and in 41 lobes, one of the observers detected a nodule

### Nodule frequency

According to the reference standard, at least one nodule was identified in 38% (27/72) (95% CI: 26–49%) of the children (Table 2). A total of 59 pulmonary nodules were found among 27 patients. In the patients with nodules, up to 5 nodules per patient were present, with an average of 2.2 nodules (59/27) per patient and most patients (11/27) with 1 nodule. Logistic regression analysis of nodule frequency per year of age gave an odds ratio of 1.089 (not significant, 95% CI: 0.945–1.255). Table 3 shows the absolute probabilities of finding a nodule per age group. Although the year of the scan had no significant effect on the presence of nodules (*P*=0.09) or on the size of the smallest nodule detected (*P*=0.90), it seems that the number of nodules detected significantly increased each year (*P*=0.01).

**Table 2:** Pulmonary nodules in children on chest CT by age

Age (y)	*n*	Number of patients with nodule(s)	Number of nodules	Maximum number of nodules per patient	Mean size (range), mm	Calcified nodules	Perifissural nodules
1	3	2	3	2	3.3 (3–4)	0	2
2	5	0	0	0			
3	8	4	12	5	3.3 (2–6)	0	0
4	3	0	0	0			
5	5	1	1	1	3.0	0	6
6	6	1	1	1	4.0	0	1
7	3	2	2	1	2.0	1	1
8	14	5	12	4	2.9 (2–4)	0	10
9	4	2	4	3	3.5 (2–6)	0	3
10	3	1	3	3	3.3 (3–4)	0	3
11	7	4	8	2	3.3 (2–5)	0	6
12	11	5	13	5	3.2 (2–5)	3	7
Total	72	27 (38%^a^)	59	5	3.2 (2–6)	4 (7%)	39 (66%)

^a^95% confidence interval 26–49%

**Table 3:** The probability (95% confidence interval [CI]) of finding nodules in a child in whom the largest nodule is ≥3 mm or ≥5 mm

Age in years	Any nodule probability (CI)		>1 nodule probability (CI)		Any nodule ≥3 mm probability (CI)		Any nodule ≥5 mm probability (CI)	
≥1–2	0.67	(0.13–1.00)	0.33	(0.00–0.87)	1.00	-	0.00	-
≥2–3	0.00							
≥3–4	0.50	(0.15–0.85)	0.25	(0.00–0.55)	0.75	(0.33–1.00)	0.00	-
≥4–5	0.00							
≥5–6	0.20	(0.00–0.55)	0.00	-	1.00	-	0.00	-
≥6–7	0.17	(0.00–0.47)	0.00	-	1.00	-	0.00	-
≥7–8	0.67	(0.13–1.00)	0.00	-	0.00	-	0.00	-
≥8–9	0.36	(0.11–0.61)	0.21	(0.00–0.43)	0.80	(0.45–1.00)	0.00	-
≥9–10	0.50	(0.01–0.99)	0.25	(0.00–0.67)	1.00	-	0.50	(0.00–1.00)
≥10–11	0.33	(0.00–0.87)	0.33	(0.00–0.87)	1.00	-	0.00	-
≥11–12	0.57	(0.21–0.94)	0.57	(0.21–0.94)	1.00	-	0.25	(0.00–0.67)
≥12–13	0.45	(0.16–0.75)	0.36	(0.08–0.65)	0.80	(0.45–1.00)	0.20	(0.00–0.55)

### Nodule characteristics

**Fig. 3:**
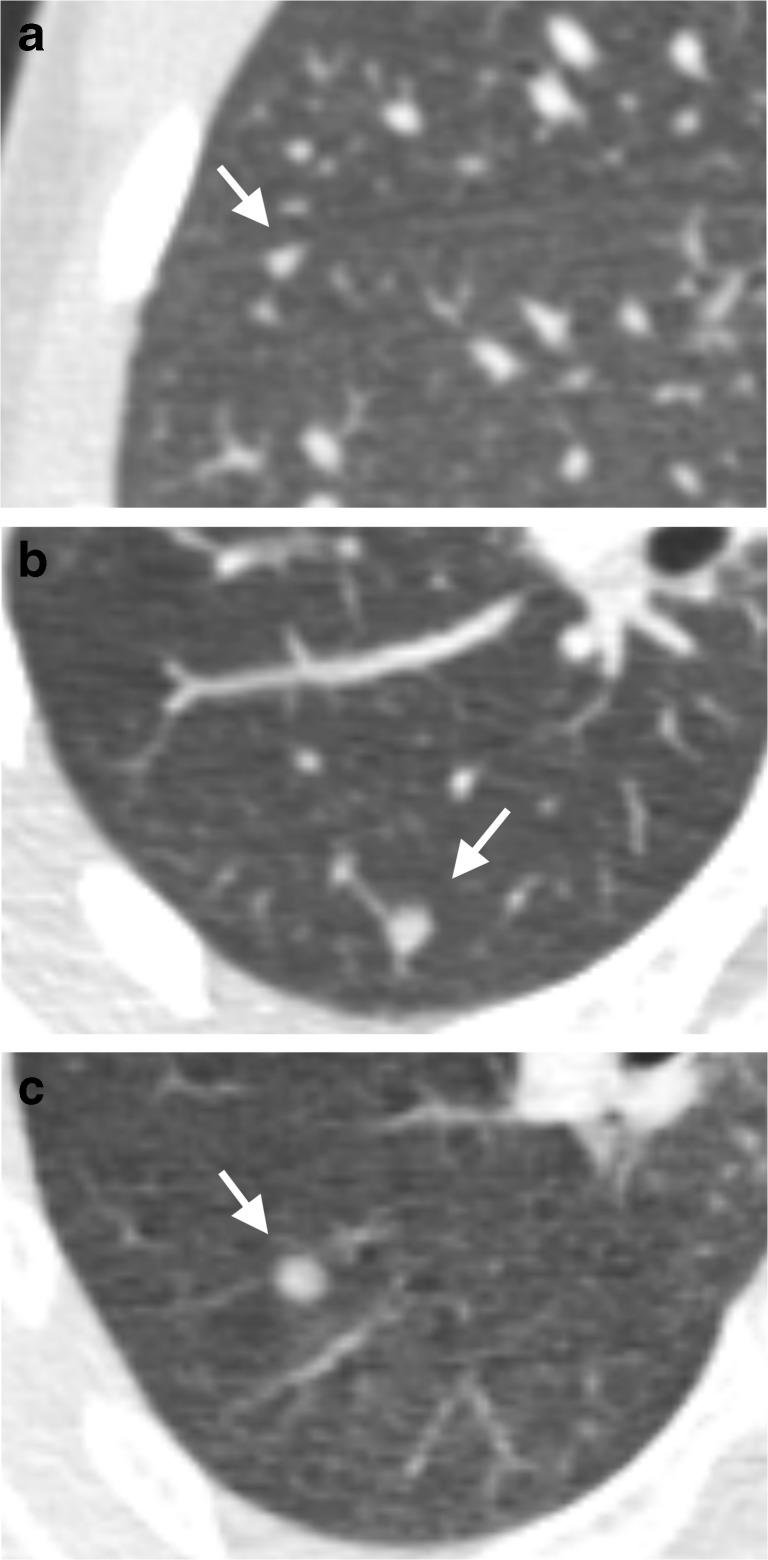
Axial 0.9-mm-thick CT images show examples from the perifissural nodule classification [14]. **a** A 3-mm typical perifissural nodule (*arrow;* fissure attached, homogenous, smooth margins and triangular shape) in an 8-year-old boy. **b** A 5-mm atypical perifissural nodule (*arrow;* fissure attached, homogenous, smooth margins and convex on one side, rounded on the other) in an 11-year-old boy. **c** A 5-mm non–perifissural nodule (*arrow;* fissure attached, inhomogenous, blurred margins and spherical shape) in a 12-year-old boy

The mean nodule size was 3.2 mm (SD: 0.9 mm) and the largest nodule measured 6 mm. Of the 59 nodules, 81% (48/59) were ≥3 mm, only 7% (4/59) contained calcification and 66% (39/59) were perifissural nodules (Fig. 3). Of the 27 children who presented with nodules, 81% (22/27) presented with larger nodules (≥3 mm), 19% (5/27) with multiple nodules (≥4 nodules), and 44% (12/27) with nodules that were neither calcified nor perifissural. Of the 16 children who had more than one nodule, 63% (10/16) had bilaterally distributed nodules. Nodules occurred with greatest frequency in the right lower lobe (31%, 18/59), followed by the right upper lobe (24%, 14/59), left lower lobe (19%, 11/59), right middle lobe (17%, 10/59) and the left upper lobe (10%, 6/59).

## Discussion

The most important findings of this study are that small pulmonary nodules are common in otherwise healthy young children, but that they can be difficult to detect. Perifissural nodules and nodules without clearly benign characteristics do occur in otherwise young healthy children. These findings can be relevant as background information for managing young children with cancer.

A study by Alves et al. [12], investigating children with pectus excavatum/carinatum, found a remarkable high nodule prevalence (75%), ranging up to 21 nodules per patient, despite excluding all nodules that resembled intrapulmonary lymph nodes. This may be related to the subtropical region that is endemic for tuberculosis, histoplasmosis and paracoccidioidomycosis. Meanwhile, a study by Renne et al. [11] in a paediatric trauma setting reported a prevalence of 33%, ranging up to 4 nodules per patient. Our results reveal a nodule frequency (38%; 95% CI:26–49%) and range (up to 5 nodules per patient) consistent with Renne et al. [11]. In the patients with nodules, the average nodule-per-patient rate (2.2) falls in between the rates of Alves et al. (3.4) [12] and Renne et al. (1.5) [11]. Hence, we confirm previous reports, suggesting that small pulmonary nodules on CT are indeed a common finding in young children without malignancy.

Renne et al. [11] found that multiple nodules (≥4 nodules) were present in a small subset (5%) of patients, which was also the case in our study (19%). Furthermore, with perifissural nodules a frequent finding in our cohort of children (66%, 39/59 nodules), it is clear that perifissural nodules are not solely limited to adults. In adults, the portion of perifissural nodules has been reported to be 23% to 28% [14, 16, 17]. Whether perifissural nodules are indeed benign in adults or children who present with a malignancy has not been investigated to our knowledge.

Our results show that, with a moderate (*κ*=0.40) interobserver agreement on a per-lobe basis, it is difficult to detect small pulmonary nodules at chest CT. This corresponds to our experience in adults, although the literature on nodules <4 mm is limited as nodules of this size are often regarded as benign in adults [4]. Regarding detection, Scholten et al. [18] reported an almost perfect (*κ*=0.89–0.95) agreement for larger nodules (mean: 11.5, SD: 3.8 mm), while Kilburn-Toppin et al. [19] found moderate (*κ*=0.42–0.50) agreement for smaller (mostly <4 mm) nodules. Focusing on children with Wilms tumour, Wilimas et al. [20] found substantial interobserver variability (*P*<0.01) in nodule detection: Of the 78 cases, 41 were rated positive by only one reader, 18 by two readers and 19 by all three readers. Overall, this underscores the fact that detecting small pulmonary nodules on CT is challenging. We found substantial agreement (*κ*=0.74) regarding agreement for size category. Little variability in size category is important because this 1-mm margin between a 2-mm and 3-mm lesion determines whether stage IV therapy is indicated in children with Wilms tumour [3]. The low agreement on perifissural yes/no (*κ*=0.36) indicates how difficult it is to classify such small nodules. As for calcification (*κ*=0.46), the readers only disagreed on 2 of the 22 nodules. We found that the small sizes of the nodules (2–3 mm) and only partially increased density made it difficult to differentiate between real calcification and artefact due to respiratory motion or beam hardening.

Our study can have clinical implications for the assessment and management of pulmonary nodules on CT. We provided normative data that adds to our understanding of incidental nodules in otherwise healthy children. Normative data is important to be able to correctly judge the probability of pulmonary nodules being metastases in children presenting with a malignancy.

The prognostic significance of small pulmonary nodules on CT in children with Wilms tumour has been studied by comparing the treatment as localized disease vs. the treatment as metastatic disease on event-free survival and overall survival [21, 22]. In one study, more intense stage IV treatment showed no significant improvement of the 3-year event-free survival, while the other study showed a significant improvement of the 5-year event-free survival, which might suggest the nodules were metastases. Nevertheless, both studies found no significant improvement in overall survival, which may imply the dissolvement of small lesions by regular chemotherapeutic regimens, or a misinterpretation of benign nodules as metastases [10]. This is important, as children diagnosed with Wilms tumour and metastasis-suspect lung nodules will receive more intense stage IV treatment, including doxorubicin with or without chest radiotherapy [3]. Our study shows that multiple nodules (≥4 nodules), larger nodules and nodules without clear benign characteristics (non–calcified, non–perifissural nodules) also occur in children without malignancy. As a result, there may be a considerable risk of incorrect upstaging, unnecessarily subjecting children with Wilms tumour to the dangers of more intense therapies, such as doxorubicin-related cardiotoxicity or radiation-induced second malignancies [23–25]. Hence, we feel that further discussion is needed on whether additional testing or follow-up is warranted before initiating more intense treatment in children with Wilms tumours presenting with a few small pulmonary nodules.

The uncertainty in diagnosis has to be stressed, as our study confirms substantial interobserver variability in detecting small pulmonary nodules and in individual interpretation of nodules. Great care has to be taken with managing patients in the setting of low validity tests. Further investigation of computer-aided detection as a first, concurrent or second reader may be worthwhile to increase detection rates,. Thus far, computer-aided detection in children has only proven to be helpful for nodules ≥4 mm, but those systems are rapidly improving [26].

Some limitations of our study may be considered. First, our relatively small sample size may have impaired the precision and statistical certainty of our results. Still, the outcome for nodule frequency was comparable to that of previous literature [11]. Second, we have to acknowledge that kappa calculation is difficult. Kappa calculation for nodule-based agreement was not possible because agreement on negative cases would have been zero. Therefore, we presented our data on a patient and lobe basis. Nevertheless, even lobe-based agreement does not necessarily mean that both observers found identical nodules in the concerning lobe. Third, unenhanced chest CT is standard for metastasis screening in children. We cannot exclude that contrast injection altered detection rates or size measurements in our study [27]. Fourth, the relatively long sampling period might have introduced heterogeneity to the results. The significant increase in nodule number each year may be the result of newer equipment, e.g., new multi-detector CT scanners with improved spatial and temporal resolution and techniques. Another limitation of our retrospective cohort study is the lack of a gold standard. Follow-up or histological confirmation of these coincidentally found nodules is not feasible due to ethical and practical reasons [28]. Although the probability of the nodules being malignant in our study is rather small [29], it is not possible to rule out malignant disease. Lastly, we want to discuss potential bias in the selection of patients. Since the majority of patients were male, potential gender bias may be considered. Ethnic background was not determined in our study but may be relevant for further studies. Although some nodules might be related to recent infections or trauma, we don’t think this substantially influenced our results. There is no real evidence that the occurrence of small subcentrimetric nodules as found in our study may be an early result of chest trauma. Miller et al. [30] noted that lacerations may appear as opacified nodules due to formed blood clots in the weeks after trauma. On the other hand, most scans in our study were made within an hour after the trauma. More frequently, the occurrence of patchy ground glass opacities surrounding contusions or cavitary lesions (traumatic pulmonary pseudocysts or post-traumatic pneumatocele) have been described [31, 32]. However, all patients with extensive posttraumatic changes have been excluded in our study.

## Conclusion

Small pulmonary nodules are a frequent finding in otherwise healthy children undergoing chest CT in a trauma setting. However, we demonstrated that multiple larger nodules without clear benign characteristics also occur in children without malignancy, with moderate interobserver agreement in nodule detection of nodules. These findings can influence the discussion on how to manage young children with cancer.
